# Post-translational Modifications and Protein Quality Control in Motor Neuron and Polyglutamine Diseases

**DOI:** 10.3389/fnmol.2017.00082

**Published:** 2017-03-31

**Authors:** Fabio Sambataro, Maria Pennuto

**Affiliations:** ^1^Department of Experimental and Clinical Medical Sciences, University of UdineUdine, Italy; ^2^Centre for Integrative Biology, Dulbecco Telethon Institute, University of TrentoTrento, Italy

**Keywords:** post-translational modifications, motor neuron disease, polyglutamine disease, protein degradation, aggregation

## Abstract

Neurodegenerative diseases, including motor neuron and polyglutamine (polyQ) diseases, are a broad class of neurological disorders. These diseases are characterized by neuronal dysfunction and death, and by the accumulation of toxic aggregation-prone proteins in the forms of inclusions and micro-aggregates. Protein quality control is a cellular mechanism to reduce the burden of accumulation of misfolded proteins, a function that results from the coordinated actions of chaperones and degradation systems, such as the ubiquitin-proteasome system (UPS) and autophagy-lysosomal degradation system. The rate of turnover, aggregation and degradation of the disease-causing proteins is modulated by post-translational modifications (PTMs), such as phosphorylation, arginine methylation, palmitoylation, acetylation, SUMOylation, ubiquitination, and proteolytic cleavage. Here, we describe how PTMs of proteins linked to motor neuron and polyQ diseases can either enhance or suppress protein quality control check and protein aggregation and degradation. The identification of molecular strategies targeting these modifications may offer novel avenues for the treatment of these yet incurable diseases.

## Introduction

**Figure 1:**
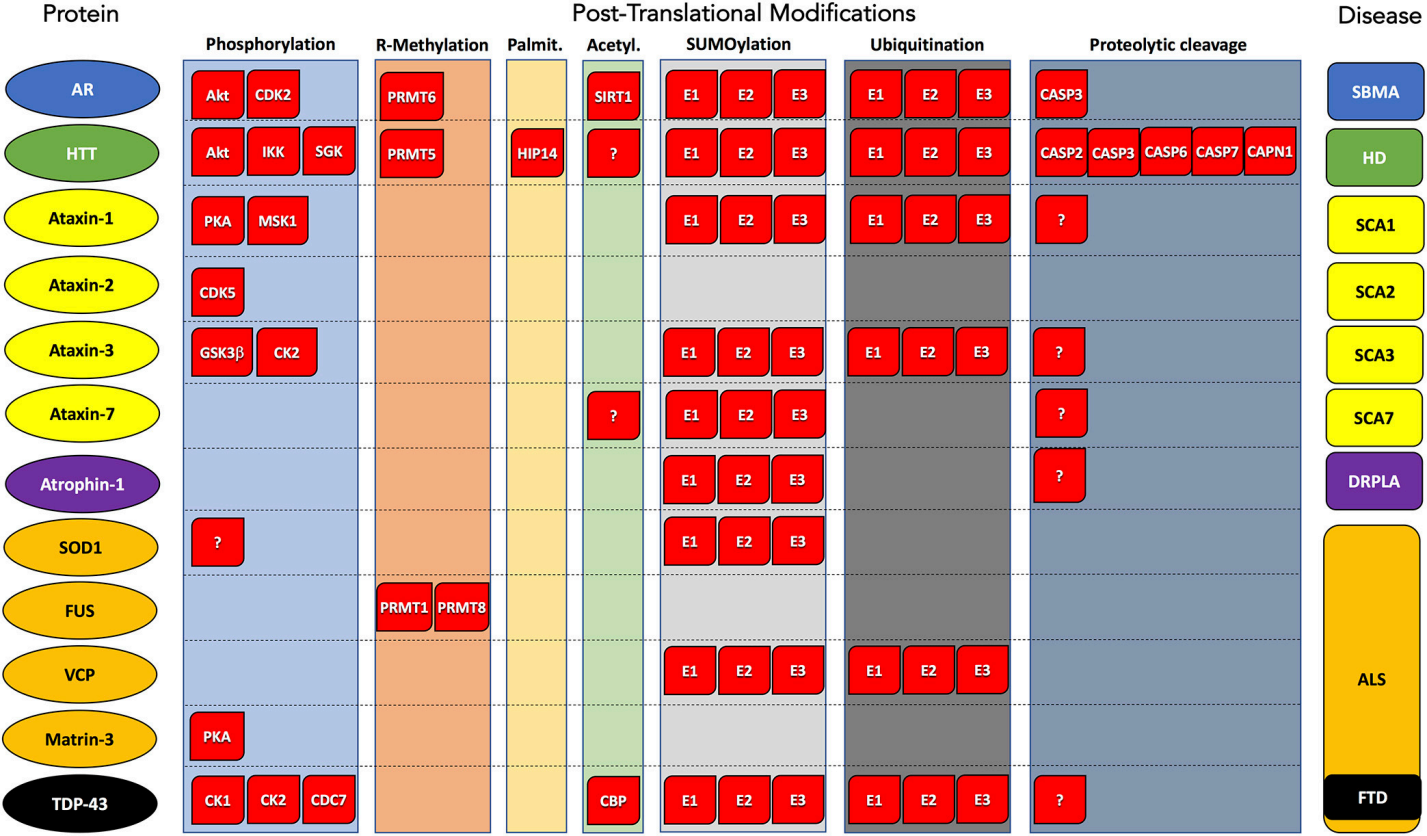
**Post-translational modifications of proteins associated with polyglutamine and motor neuron diseases**. Substrate protein, PTM type, and corresponding disease are reported by column. Enzyme names are indicated within red boxes. AR, Androgen Receptor; HTT, Huntigtin; ATX1, Ataxin 1; ATX2, Ataxin 2; ATX3, Ataxin 3; ATX7, Ataxin 7; SOD1, superoxide dismutase 1; FUS, FUS RNA-binding protein; VCP, valosin-containing protein; TDP-43, TAR DNA-binding protein 43; ATN1, atrophin 1. SBMA, Spinal and bulbar muscular atrophy; HD, Huntington's disease; SCA1, Spinocerebellar ataxia 1; SCA2, Spinocerebellar ataxia 2; SCA3, Spinocerebellar ataxia 3; SCA7, Spinocerebellar ataxia 7; ALS, Amyotrophic lateral sclerosis; FTD, Frontotemporal dementia; DRLPA, Dentatorubral-pallidoluysian atrophy.

Neurodegenerative diseases are a family of neurological disorders that includes Alzheimer's disease, Parkinson's disease, motor neuron diseases, and polyglutamine (polyQ) diseases (reviewed by Dugger and Dickson, 2017). These diseases are characterized by a variety of motor, cognitive, and behavioral symptoms. Typically neurodegenerative diseases are late-onset and manifest with progressively deteriorating phenotypes. Most diseases are sporadic, and only a limited number of cases is familial. Causal genes have been identified only for few neurodegenerative diseases. Specific populations of neurons become dysfunctional and eventually die leading to different cognitive and motor symptoms. Neurodegenerative diseases are mainly associated with toxic gain of function mechanisms that lead to the alteration of several cellular pathways. A hallmark of neurodegenerative diseases is the accumulation of abnormally folded proteins in the forms of inclusions and micro-aggregates both inside and outside the neurons. Due to the presence of these insoluble species, these disorders are also known as brain folding diseases or proteinopathies. Abnormal species, which are found both in the nucleus and in the cytoplasm, can acquire amyloid-like properties, and move from one neuronal cell to another along anatomically and functionally interconnected areas in the central nervous system. Depending on the aethiologic agent that forms aggregates and on the type of aggregate, brain folding diseases can be subdivided into four types of proteinopathies, namely amyloidoses, tauopathies, α-synucleinopathies, and transactivation response-DNA binding protein 43 (TDP-43) proteinopathies (reviewed by Dugger and Dickson, 2017). TDP-43 proteinopathies include sporadic frontotemporal lobar degeneration with tau-negative and ubiquitin-positive inclusions (FTLD-U) with and without motor neuron disease (MND), familial forms of FTLD-U with mutations in the progranulin gene (GRN), valosin-containing protein (VCP), and linkage to chromosome 9p, as well as most forms of amyotrophic lateral sclerosis (ALS) except for familial ALS with Cu/Zn superoxide dismutase 1 (SOD-1) mutations. Although sporadic in most cases, familial forms of ALS have been associated with mutations in specific genes, including SOD1, TDP-43, fused in sarcoma/translocated in liposarcoma (FUS/TLS, which we will refer to hereafter as FUS), VCP, optineurin, tank-binding kinase 1 (TBK1), dynactin subunit 1, angiogenin, C9orf72, ubiquilin 2, sequestosome 1 (p62), profiling-1, hnRNP A1, matrin-3, tubulin α-4A chain, and coiled-coil-helix-coiled-coil-helix domain-containing protein 10 (reviewed by Taylor et al., 2016). The deposition of misfolded proteins in inclusions and micro-aggregates is also an important feature of polyQ diseases, which are caused by expansions of CAG trinucleotide tandem repeats encoding glutamine (reviewed by Orr and Zoghbi, 2007; Pennuto and Sambataro, 2010). PolyQ diseases include spinal and bulbar muscular atrophy (SBMA), Huntington's disease (HD), dentatorubral-pallidoluysian atrophy (DRPLA), and spinocerebellar ataxia (SCA) type 1, 2, 3, 6, 7, and 17. These disorders are caused by polyQ expansions in androgen receptor (AR), huntingtin (HTT), atrophin-1, ataxin-1, ataxin-2, ataxin-3, CACNA1A, ataxin-7, and the TATA-binding protein, respectively. Whether micro-aggregates and inclusions are toxic or protective species and participate in disease pathogenesis or represent an adaptive response to proteotoxic stress remains controversial. Indeed, neurons expressing mutant HTT and AR and capable of forming inclusions survive longer compared to those unable to produce inclusions (Arrasate et al., 2004; Palazzolo et al., 2010). This observation suggests their role as protective species that allow the confinement of toxic proteins to subcellular areas dedicated to protein clearance by the ubiquitin-proteasome system (UPS) and macro-autophagy (hereafter referred to as autophagy). Because neurodegenerative diseases are associated with the accumulation of toxic species that cause neurodegeneration through toxic gain of function mechanisms, the clearance of the disease-causing proteins is key to fight the neurodegenerative processes. Recent emerging evidence has shown that post-translational modifications (PTMs) of proteins linked to neurodegenerative diseases can affect their subcellular localization, activity, protein-protein interaction, stability, aggregation, and clearance by the UPS and autophagy. Here, we review the PTMs capable of affecting the aggregation, the inclusion formation, the turnover and the degradation of proteins linked to polyQ and motor neuron diseases (Figure 1, Table 1).

**Table 1:** **Post-translational modifications (PTMs) of proteins linked to polyglutamine and motor neuron diseases**.

**Protein**	**Site**	**Kinase**	**Effect on disease**	**Mechanism**
**PHOSPHORYLATION**				
AR	^210^RXRXXS^215^, ^787^RXRXXS^792^	Akt	Protective	Enhanced degradation by UPS, reduced aggregation (Palazzolo et al., 2007, 2009; Nedelsky et al., 2010; Rinaldi et al., 2012)
AR	S96	CDK2	Toxic	Increased protein stability (Polanco et al., 2016)
HTT	^416^RXRXXS^421^	Akt, SGK	Protective	Enhanced degradation by UPS (Humbert et al., 2002; Rangone et al., 2004; Warby et al., 2005; Kratter et al., 2016)
HTT	S13/S16	IKK	Protective	Enhanced degradation by UPS and autophagy (Gu et al., 2009; Thompson et al., 2009)
HTT	T3		Protective	Increased inclusion formation (Aiken et al., 2009)
Ataxin-1	^771^RXRXXS^776^	PKA, MSK1	Toxic	Increased protein stability (Chen et al., 2003; Jorgensen et al., 2009; Park et al., 2013)
Ataxin-2		CDK5		Enhanced degradation (Asada et al., 2014)
Ataxin-3	S256	GSK3β	Protective	Decreased aggregation (Fei et al., 2007)
Ataxin-3	S236, S256, S260, S261, S340, S352	CK2		Increased protein stability and nuclear inclusion formation (Tao et al., 2008; Mueller et al., 2009)
Ataxin-3	S12			Increased protein stability and nuclear inclusion formation (Matos et al., 2016)
TDP-43	S379, S403, S404, S409, S410	CK1, CK1δ, CK1ε, CK2, CDC7	Toxic	Enhanced aggregation, decreased degradation (Neumann et al., 2006; Hasegawa et al., 2008; Inukai et al., 2008; Neumann et al., 2009; Liachko et al., 2010; Zhang et al., 2010; Brady et al., 2011; Li et al., 2011; Liachko et al., 2013; Choksi et al., 2014; Liachko et al., 2014, 2016)
SOD1	T2		Protective	Decreased aggregation (Fay et al., 2016)
Matrin-3		PKA		Enhanced degradation (Giordano et al., 2005)
**ARGININE METHYLATION**				
AR	^210^RXRXXS^215^, ^787^RXRXXS^792^	PRMT6	Toxic	Reduced phosphorylation by Akt, increased aggregation (Scaramuzzino et al., 2015)
FUS		PRMT1, PRMT8	Protective	Regulation of subcellular localization (Rappsilber et al., 2003; Kim et al., 2008; Jobert et al., 2009; Du et al., 2011; Dormann et al., 2012; Tradewell et al., 2012; Scaramuzzino et al., 2013; Tibshirani et al., 2015; Fujii et al., 2016)
**PALMITOYLATION**				
HTT		HIP14	Protective	Decreased inclusion formation (Yanai et al., 2006)
**ACETYLATION**				
AR	^630^KXKK^633^	SIRT1	Toxic	Enhanced aggregation and nuclear localization (Lieberman et al., 2002; Nedelsky et al., 2010; Palazzolo et al., 2010; Montie et al., 2011)
HTT	K6, K9, K15		Protective	Decreased aggregation, enhanced degradation by UPS (Jia et al., 2012; Yue et al., 2015; Chaibva et al., 2016)
HTT	K444		Protective	Enhanced degradation by autophagy (Jeong et al., 2009)
Ataxin-7	K257		Toxic	Decreased clearance (Mookerjee et al., 2009)
TDP-43	K145, K192	CBP	Toxic	Increased aggregation (Cohen et al., 2015)
**SUMOylation**				
AR	K385, K518		Toxic	Mukherjee et al., 2009; Chua et al., 2015
HTT	K6, K9, K15		Toxic	Increased protein stability (Steffan et al., 2004)
Atrophin-1				Enhanced aggregation (Terashima et al., 2002)
Ataxin-1	K16, K194, K610, K697, K746		Toxic	Enhanced aggregation (Riley et al., 2005; Ryu et al., 2010)
Ataxin-3	K356			Decreased aggregation (Almeida et al., 2015)
Ataxin-3	K166		Toxic	Increased protein stability (Zhou et al., 2013)
Ataxin-7	K257		Protective	Decreased aggregation (Janer et al., 2010)
VCP			Protective	Enhanced degradation of proteins via ERAD (Wang et al., 2016)
SOD1	K9, K75		Toxic	Increased protein stability and aggregation (Fei et al., 2006; Niikura et al., 2014; Dangoumau et al., 2016)
TDP-43				Enhanced aggregation (Seyfried et al., 2010)
**UBIQUITINATION**				
AR		CHIP	Protective	Decreased aggregation (Adachi et al., 2007)
HTT	K6, K9, K15		Protective	Enhanced degradation (Jana et al., 2005)
HTT		CHIP	Protective	Decreased aggregation (Jana et al., 2005)
Ataxin-1		CHIP	Protective	Decreased aggregation (Al-Ramahi et al., 2006)
Ataxin-3		CHIP, Parkin	Protective	Decreased aggregation (Matsumoto et al., 2004; Jana et al., 2005; Warrick et al., 2005)
TDP-43		Parkin	Protective	Enhanced degradation (Neumann et al., 2006; Hebron et al., 2013, 2014)
**PROTEOLYTIC CLEAVAGE**				
AR		Caspases	Toxic	Enhanced aggregation (Wellington et al., 1998)
HTT		Caspases, Calpains	Toxic	Enhanced aggregation (Goldberg et al., 1996; Wellington et al., 1998, 2000; Kim et al., 2001; Gafni and Ellerby, 2002; Lunkes et al., 2002; Gafni et al., 2004; Hermel et al., 2004)
Atrophin-1		Caspases	Toxic	Enhanced aggregation (Miyashita et al., 1997; Wellington et al., 1998)
Ataxin-3		Caspases	Toxic	Enhanced aggregation (Berke et al., 2004; Haacke et al., 2006)

*Amino acid and site are reported. Consensus site sequence is also included when available*.

## Phosphorylation

Phosphorylation is regulated by the balance between the activity of cellular kinases and phosphatases, whose function is modulated by specific cellular pathways. In disease conditions, alteration of these cellular pathways can result in dysregulation of kinase and phosphatase activity and aberrant PTM of the target proteins. Several proteins linked to neurodegenerative diseases are aberrantly (hyper- or hypo-) phosphorylated in pathological conditions. Phosphorylation affects the homeostasis of these proteins thus modifying—either suppressing or enhancing—toxicity. There is remarkable evidence that phosphorylation modifies the toxicity of polyQ-expanded proteins and proteins linked to motor neuron diseases.

### Spinal and bulbar muscular atrophy

Phosphorylation of polyQ-expanded proteins can either increase protein stability, thereby enhancing neuronal dysfunction and death, or promote the clearance of the disease protein through the UPS and autophagy, which leads to neuroprotection. Normal AR and polyQ-expanded AR are substrates of protein kinase B (also known as Akt; Lin et al., 2001; Palazzolo et al., 2007). Phosphorylation of AR at serine 215 (S215) and S792 within the Akt consensus sites RXRXXS (where R is arginine and X any amino acid) by Akt induces the degradation of the protein by the UPS. The activation of Akt and phosphorylation of polyQ-expanded AR at S215 and S792 reduces neurotoxicity in cell, fly, and mouse models of SBMA (Palazzolo et al., 2007, 2009; Nedelsky et al., 2010). Cyclin-dependent kinase 2 (CDK2) is another kinase that affects AR stability and degradation. Phosphorylation of polyQ-expanded AR at S96 by CDK2 stabilizes the disease protein and increases aggregation and neurotoxicity, suggesting a role for CDK2 in SBMA pathogenesis (Polanco et al., 2016).

### Huntington's disease

Phosphorylation of polyQ-expanded HTT at S421 within the Akt consensus site RXRXXS by Akt and serum- and glucocorticoid-induced kinase (SGK) reduces inclusion formation and neurotoxicity in HD (Humbert et al., 2002; Rangone et al., 2004), and is negatively regulated by the protein phosphatase, calcineurin (Pardo et al., 2006). Phosphorylation of HTT at S421 is halted by polyQ expansion (Warby et al., 2005; Pardo et al., 2006). Recently, phosphorylation of polyQ-expanded HTT at S421 has been shown to promote the clearance of the disease protein by the UPS, further supporting the concept that phosphorylation is a means to promote disease protein disposal and suppress neurotoxicity (Kratter et al., 2016). Since several proteins causing neurodegenerative diseases have canonical Akt consensus sites, it is possible that phosphorylation by Akt modifies protein homeostasis and toxicity in other neurodegenerative diseases (Basso and Pennuto, 2015). The phosphorylation of HTT at S13 and S16, which is regulated by the inflammatory kinase IκB kinase (IKK), enhances HTT clearance through the UPS and autophagy and reduces aggregation, thus protecting from neurodegeneration (Gu et al., 2009; Thompson et al., 2009). Phosphorylation at those sites is reduced by polyQ-expansion, suggesting that aberrant phosphorylation of polyQ-expanded HTT contributes to disease pathogenesis. Moreover, polyQ expansion reduces the phosphorylation of polyQ-expanded HTT at threonine 3 (T3; Aiken et al., 2009), a site where phosphorylation increases inclusion formation and aggregation and ultimately reduces neurodegeneration.

### Spinocerebellar ataxia

Phosphorylation of polyQ-expanded ataxin-1 at S776 increases its binding to the molecular chaperone 14-3-3, which enhances protein stability and neurodegeneration (Chen et al., 2003). Ataxin-1 is phosphorylated at S776 by protein kinase A (PKA) and mitogen- and stress-activated protein kinase-1 (MSK1), and stimulation of pathways, such as the RAS/MAPK-MSK1 pathway, that leads to the activation of these kinases enhances the accumulation of the mutant protein and neurodegeneration (Jorgensen et al., 2009; Park et al., 2013). Ataxin-2 phosphorylation by CDK5 leads to the degradation of the protein by the UPS, suggesting that activation of CDK5 may reduce toxicity in SCA2 (Asada et al., 2014). PolyQ-expanded ataxin-3 is phosphorylated by glycogen synthase kinase 3 beta (GSK3β) at S256, and this phosphorylation is decreased by polyQ expansion (Fei et al., 2007). Importantly, phosphorylation of ataxin-3 by GSK3β decreases aggregation, suggesting a protective effect in SCA3. Phosphorylation of ataxin-3 at multiple sites (S236, S256, S260, S261, S340, S352) by protein casein kinase 2 (CK2) increases nuclear localization, protein stability and nuclear inclusion formation (Tao et al., 2008; Mueller et al., 2009). These observations indicate that phosphorylation at the same site, S256, by different kinases, i.e., GSK3β and CK2, may have different consequences in the cells depending on cellular context and on the different cellular pathways activated. Recently, ataxin-3 has been shown to be phosphorylated at S12, a phosphorylation event that reduces aggregation as well as dendrite and synapse loss (Matos et al., 2016). Interestingly, profilin has been reported to reduce aggregation of polyQ-expanded HTT and AR, and this function is inhibited upon phosphorylation of profilin by the Rho-associated kinase ROCK at S137 (Shao et al., 2008).

### TDP-43 proteinopathies

TDP-43 is hyperphosphorylated in TDP-43 proteinopathies (Neumann et al., 2006). This protein is phosphorylated at S379, S403, S404, S409, and S410 (Inukai et al., 2008; Neumann et al., 2009). Phosphorylation at these sites can be detected in brain specimens derived from individuals affected by FTLD-U and ALS but not from normal subjects, and positively correlates with accumulation of aggregation-prone TDP-43 fragments (Neumann et al., 2006; Hasegawa et al., 2008; Brady et al., 2011). Casein kinase 1 (CK1), CK1δ, CK1ε, and cell division cycle 7 (CDC7) have been shown to phosphorylate TDP-43 (Hasegawa et al., 2008; Liachko et al., 2013, 2014; Choksi et al., 2014). In particular, the phosphorylation at S409/410 decreases protein degradation, enhances aggregation and results in neurodegeneration (Hasegawa et al., 2008; Liachko et al., 2010, 2013; Zhang et al., 2010; Brady et al., 2011). Furthermore, hyperphosphorylation at S409/410 was observed in *C. elegans* models of TDP-43 proteinopathies, and substitution of these serines with non-phosphorylatable alanine prevented degeneration, indicating that phosphorylation at these sites is toxic (Liachko et al., 2010). On the other hand, phospho-mimetic substitution of S409/410 with aspartic acid has been shown to reduce aggregation and to enhance the clearance of the protein by autophagy and to a minor extent by UPS, suggesting that phosphorylation at specific sites may represent a self-defense mechanism to promote disposal of aggregation-prone proteins in TDP-43 proteinopathies (Brady et al., 2011). Recently, phosphorylation of this protein has been shown to be negatively regulated by calcineurin, whose activity reduces TDP-43 accumulation and neurodegeneration (Liachko et al., 2016). An alternative finding is that phosphorylation of TDP-43 by CK2 decreases aggregation (Li et al., 2011). These observations suggest that phosphorylation of TDP-43 may have different effects depending on the kinase and signaling pathways that stimulate phosphorylation of the protein at a specific site and cell context in which the phosphorylation event takes place.

### SOD1-linked amyotrophic lateral sclerosis

Similarly, phosphorylation modifies also aggregation and neurotoxicity in SOD1-linked ALS. Phosphorylation of mutant SOD1 at T2 promotes the acquisition of the native conformation and protects from neurodegeneration, indicating that phosphorylation affects protein structure and toxicity (Fay et al., 2016). It is interesting to note that TDP-43 phosphorylation is increased in mouse models of SOD1-linked ALS, suggesting that TDP-43 is involved also in the pathogenesis of ALS in patients carrying mutations in the gene coding for SOD1 (Cai et al., 2015). Activation of N-methyl-D-aspartate (NMDA) receptors leads to PKA-mediated phosphorylation of matrin-3 and degradation (Giordano et al., 2005). As inhibition of PKA prevents NMDA receptor-induced cell death and PTM of matrin-3, this pathway might be relevant also for the forms of ALS associated with mutations in matrin-3.

## Arginine methylation

Arginine methylation is emerging as a key PTM in neurodegenerative diseases. Arginine methylation is catalyzed by a class of enzymes, known as protein arginine methyltransferases (PRMTs; reviewed by Blanc and Richard, 2017). These enzymes catalyze the addition of either one methyl group to generate monomethylarginine, or of two methyl groups to generate asymmetric (type I) and symmetric (type II) dimethylarginine. To date, at least 11 PRMTs have been identified. Although enzymes with arginine demetylase activity have not yet been identified, it is possible that arginine methylation is reversible. Similar to phosphorylation, the observation that arginine methylation modifies neurotoxicity in polyQ and motor neuron diseases has therapeutic relevance. Understanding the role of PRMTs in neurodegenerative diseases and discovery of enzymes that catalyze the reverse reaction may lead to identification of novel therapeutic targets for these disorders.

### Spinal and bulbar muscular atrophy

PRMT6 is a type I enzyme that interacts with the AR, and this interaction is enhanced by polyQ expansion (Scaramuzzino et al., 2015). PRMT6 methylates the arginine residues at the Akt consensus sites (RXRXXS) of polyQ-expanded AR. Importantly, arginine methylation by PRMT6 and phosphorylation by Akt are mutually exclusive. Arginine methylation enhances polyQ-expanded AR aggregation and counteracts the protective effects of phosphorylation by Akt. These observations highlight the interplay between different PTMs with opposite effects on neurotoxicity. Because several proteins involved in neurodegenerative diseases bear one or more Akt consensus sites, alterations in the balance between phosphorylation by Akt and arginine methylation by the PRMTs could have major effects on neurotoxicity not only in SBMA, but also in other neurodegenerative diseases (Basso and Pennuto, 2015).

### Huntington's disease

Huntingtin has been shown to interact with PRMT5, but whether HTT is a substrate of PRMT5 remains to be established (Ratovitski et al., 2015).

### FUS-linked amyotrophic lateral sclerosis and FTLD

Wild type and mutant FUS undergo extensive arginine methylation (Kim et al., 2008; Jobert et al., 2009; Du et al., 2011). Wild type and mutant FUS form a complex with PRMT1 and PRMT8, which are responsible for asymmethic dimethylation of the protein (Rappsilber et al., 2003; Tradewell et al., 2012; Scaramuzzino et al., 2013). Arginine methylation regulates its subcellular localization (Araya et al., 2005; Jobert et al., 2009; Du et al., 2011; Tradewell et al., 2012; Scaramuzzino et al., 2013; Suarez-Calvet et al., 2016). Inhibition of PRMT1 in motor neuron cultures results in sequestration of FUS to nucleus with reduced stress granule formation (Tradewell et al., 2012). Nuclear localization of FUS is regulated by transportin. Arginine methylation has been shown to reduce binding to transportin and nuclear import, suggesting a pathogenic role for this PTM in FUS-linked ALS (Dormann et al., 2012). On the other hand, pharmacologic inhibition of arginine methylation by treatment with adenosine dialdehyde (AdOx) reduces the cytosolic localization and aggregation of FUS, likely reflecting cell type-specific effects of FUS arginine methylation (Scaramuzzino et al., 2013; Fujii et al., 2016). PRMT1 and PRMT8 are sequestered into stress granules, leading to the loss of nuclear PRMT1 function and altered arginine methylation of PRMT1 substrates, including histone 4 arginine 3 methylation, and dysregulation of gene expression (Scaramuzzino et al., 2013; Tibshirani et al., 2015). Consistent with the idea that loss of PRMT function may play a role in disease pathogenesis, knocking down PRMT1 exacerbates neurodegeneration in fly models of FUS-related ALS (Scaramuzzino et al., 2013). Notably, FUS arginine methylation differs between FUS-linked FTLD and FUS-linked ALS, with monomethylated FUS being the predominant species in FUS-linked FTLD, but not FUS-linked ALS. This finding highlights major differences in the pathogenic pathways that regulate arginine methylation in these two diseases (Suarez-Calvet et al., 2016).

## Palmitoylation

Palmitoylation is the covalent attachment of a saturated palmitic fatty acid chain to a cysteine residue of proteins. This reversible reaction is catalyzed by palmitoyltransferases. Palmitoylation regulates the trafficking and function of several proteins, including HTT. PolyQ expansion in HTT reduces interaction with the huntingtin interacting protein 14 (HIP14), resulting in decreased HTT palmitoylation, increased inclusion formation, and enhanced neurotoxicity (Yanai et al., 2006). These observations indicate that palmitoylation modifies HD pathogenesis. The role of palmitoylation in the pathogenesis of other polyQ diseases and motor neuron diseases is not known.

## Acetylation

Acetylation, which consists of the covalent binding of an acetyl group to a lysine (K) residue of a protein, is operated by histone acetyltransferase (HAT) enzymes. The reaction is reversible, and removal of the acetyl groups is catalyzed by histone deacetylase (HDAC) enzymes. There is a large body of evidence showing that acetylation modifies the pathogenesis of polyQ and motor neuron diseases.

### Spinal and bulbar muscular atrophy

PolyQ-expanded AR is aberrantly acetylated in motor neuron-derived MN-1 cells (Lieberman et al., 2002). AR is acetylated at ^630^KXKK^633^ (where K is lysine) motif by the NAD-dependent sirtuin, SIRT1 (Fu et al., 2000; Montie et al., 2011). The loss of acetylation at this site reduces the nuclear localization of polyQ-expanded AR, enhances AR transactivation, decreases aggregation, and protects from neurodegeneration, indicating that acetylation is a key PTM in SBMA (Nedelsky et al., 2010; Palazzolo et al., 2010; Montie et al., 2011).

### Huntington's disease

Acetylation of HTT at K444 promotes clearance by autophagy and is neuroprotective in HD (Jeong et al., 2009). The inhibition of HDAC activity has been proven to reduce aggregation, to induce clearance by the UPS and autophagy, and to protect from neurodegeneration, and these effects are associated with increased acetylation at K9 and phosphorylation at S16 and T3 (Jia et al., 2012; Yue et al., 2015). Interestingly, acetylation of HTT at K6, K9, and K15 reduces fibrillar aggregation while promoting globular aggregation and induces binding to lipids, indicating that acetylation affects not only aggregation, but also the interaction with lipid bilayers (Chaibva et al., 2016).

### Spinocerebellar ataxia

Acetylation of ataxin-7 at K257 decreases protein turnover by macroautophagy, leading to the accumulation of a neurotoxic caspase 7 proteolytic cleavage fragment (Mookerjee et al., 2009).

### TDP-43 proteinopathies

TDP-43 is acetylated by the cAMP response element-binding protein (CREB)-binding protein (CBP) at K145 and K192, which lie in the two RNA recognition motifs (RRM1 and RRM2; Cohen et al., 2015). Acetylation does not affect the stability of the protein, but rather increases aggregation along with reducing binding to RNA. This PTM is induced by oxidative stress and is negatively regulated by HDAC6.

## SUMOylation

SUMOylation consists in the attachment of the SUMO (small ubiquitin-like modifier) proteins to K residues of a target protein within the consensus sequence ΨKX[D/E] (where Ψ is a hydrophobic amino acid, D aspartate, and E glutamate; reviewed by Flotho and Melchior, 2013). However, several proteins without such motif are SUMOylated. There are four SUMO proteins, namely SUMO1, SUMO2, SUMO3, and SUMO4, that are linked to the substrate in a three-step reaction regulated by a pathway analogous, but distinct from ubiquitylation. Covalent attachment of SUMO proteins is catalyzed by enzymes with SUMO E1 activating, SUMO E2 conjugating, and SUMO E3 ligase activities. SUMOylation predominantly occurs in the nucleus and can be reversed by the SUMO-specific protease family of enzymes with isopeptidase activity. PolyQ proteins as well as proteins linked to ALS are SUMOylated.

### Spinal and bulbar muscular atrophy

AR is SUMOylated at K385 and K518 (Mukherjee et al., 2009). Enhancement of AR SUMOylation has been reported to reduce aggregate formation in cultured cells (Mukherjee et al., 2009). Although the loss of AR SUMOylation ameliorates the phenotype of knock-in SBMA mice, no effect on inclusion and aggregate formation has been found *in vivo* (Chua et al., 2015).

### Huntington's disease

SUMOylation of HTT at K6, K9, and K15 is mutually exclusive with ubiquitination (Steffan et al., 2004). SUMOylation stabilizes mutant HTT, but decreases aggregation. Importantly, polyQ-expanded HTT SUMOylation enhances neurotoxicity, whereas ubiquitination has the opposite effect.

### Dentatorubral-pallidoluysian atrophy

SUMO modification increases the aggregation of polyQ-expanded atrophin-1 (Terashima et al., 2002).

### Spinocerebellar ataxia

Ataxin-1 is SUMOylated at K16, K194, K610, K697, and K746, and SUMOylation is reduced by polyQ expansion (Riley et al., 2005). Interestingly, SUMOylation is restored to normal levels by the loss of phosphorylation at S776. SUMOylation of ataxin-1 increases aggregation, is enhanced by oxidative stress and is regulated by c-Jun N-terminal kinase (JNK) signaling (Ryu et al., 2010). SUMOylation of ataxin-3 at K166 enhances protein stabilization and toxicity without affecting aggregation (Zhou et al., 2013), whereas SUMOylation at K356 decreases aggregation (Almeida et al., 2015). Ataxin-7 is SUMOylated at K257, and this PTM decreases aggregation and toxicity (Janer et al., 2010).

### Amyotrophic lateral sclerosis

SUMOylation is also involved in ALS pathogenesis. VCP is SUMOylated, and SUMOylation is decreased by pathogenic mutations (Wang et al., 2016). Under oxidative stress and endoplasmic reticulum (ER) stress SUMOylation promotes VCP hexamer formation, which is key to its ATPase activity, enhances accumulation of VCP to the nucleus and stress granule formation, and facilitates the degradation of polyubiquitinated proteins via endoplasmic reticulum-associated protein degradation (ERAD), thus implying a role for SUMOylation in protein quality control systems operating under stress conditions. SOD1 is SUMOylated at K9 and K75 (Fei et al., 2006; Niikura et al., 2014; Dangoumau et al., 2016). SUMO-1 localizes to mutant SOD1-positive aggregates. SUMOylation regulates SOD1 stability and aggregation. The loss of SUMOylation at K75 reduces SOD1 aggregation, whereas the overexpression of SUMO-1 and SUMO-3 enhances SOD1 SUMOylation, protein stabilization and aggregation. TDP-43 is SUMOylated (Seyfried et al., 2010). Overexpression of TDP-43 results in increased SUMO-2/3 accumulation in the insoluble proteome, and SUMO-2/3 localize to TDP-43-positive inclusions. These results suggest that SUMOylation contributes to TDP-43 aggregation.

## Ubiquitination

The binding of ubiquitin to K residues of a protein involves three classes of enzymes with ubiquitin-activating enzyme (E1), ubiquitin-conjugating enzyme (E2), and ubiquitin-protein ligase (E3) activities. Ubiquitination is reversible, and the removal of the ubiquitin chain is catalyzed by enzymes with de-ubiquitinating activities. Polyubiquitination is a signal for degradation mostly through the UPS, but also via autophagy. Inclusions contain ubiquitin and ubiquitinated proteins, which may represent species confined to subcellular compartments destined to degradation through the UPS and autophagy (DiFiglia et al., 1997). On the other hand, sequestration of ubiquitin and proteasome components into inclusions can alter the protein quality control machinery contributing to neurodegeneration (Donaldson et al., 2003).

### PolyQ diseases

Because of its role in protein degradation, ubiquitination is a PTM with a major role in polyQ and motor neuron diseases. Overexpression of ubiquitin ligases, such as C-terminus of Hsp70- interacting protein (CHIP) and E4B, increases the ubiquitination, decreases the aggregation, and attenuates the toxicity of polyQ proteins, such as AR, HTT, ataxin-1, and ataxin-3 (Matsumoto et al., 2004; Jana et al., 2005; Al-Ramahi et al., 2006; Adachi et al., 2007). Similarly, overexpression of the E3 ubiquitin ligase parkin induces ataxin-3 degradation and reduces aggregation and toxicity (Tsai et al., 2003). Ataxin-3 is itself a poly-ubiquitin-binding protein and its overexpression promotes polyQ protein degradation and suppresses neurodegeneration (Warrick et al., 2005). Phosphorylation affects ubiquitination of proteins linked to polyQ diseases. Phosphorylation of ataxin-1 at S776 modulates its ubiquitination by CHIP and aggregation (Choi et al., 2007).

### TDP-43 proteinopathies

TDP-43 is ubiquitinated in ALS and FTD-U brain specimens (Neumann et al., 2006).

Ubiquilin 2 binds to ubiquitinated proteins to facilitate their delivery to proteasome. Mutations causing ALS interfere with the ability of the protein to deliver cargoes to proteasome (Chang and Monteiro, 2015). Interestingly, ubiquilin 1 enhances TDP-43 aggregation and colocalization with autophagy markers (Kim et al., 2009). Parkin has been shown to ubiquitinate TDP-43, resulting in the accumulation of TDP-43 into the cytosol (Hebron et al., 2013), and reduction of toxicity (Hebron et al., 2014). On the other hand, mutations of TDP-43 have been shown to enhance ubiquitination. Since suppression of ubiquitination enhances neurodegeneration *in vivo*, this finding suggests that ubiquitination is a process involved in disease pathogenesis (Hans et al., 2014). Phosphorylation also affects ubiquitination of proteins linked to motor neuron diseases. In the response of cells to viral infections, the dual-specificity tyrosine-(Y)-phosphorylation-regulated kinase 2 (DYRK2) phosphorylates TBK1 at S527, leading to polyubiquitination and degradation of the protein (An et al., 2015). Whether this pathway plays a role in ALS associated with mutations in TBK1 remains to be established.

## Proteolytic cleavage

Proteolytic cleavage of proteins linked to polyQ diseases and motor neuron diseases is emerging as a key PTM in neurodegeneration. Indeed, short fragments of the proteins associated with these diseases have been shown to be more toxic than the full length protein, for their tendency to accumulate in the cells and to form aggregates. Animal models expressing fragments of polyQ-expanded proteins develop a more severe phenotype compared with animals expressing the full length protein, supporting the idea that small fragments are extremely toxic species.

### PolyQ diseases

Several polyQ-expanded proteins are substrates of caspases, such as HTT (Goldberg et al., 1996; Wellington et al., 1998; Kim et al., 2001), AR (Wellington et al., 1998), atrophin-1 (Miyashita et al., 1997; Wellington et al., 1998), and ataxin-3 (Berke et al., 2004; Haacke et al., 2006), or calpain, such as HTT (Kim et al., 2001; Lunkes et al., 2002). PolyQ-expanded AR is proteolytically cleaved at aspartate 146 by caspase 3, thus resulting in toxic fragments and aggregation (Ellerby et al., 1999b). Interestingly, AR cleavage is enhanced by phosphorylation at S514, indicating that phosphorylation modulates proteolytic cleavage of polyQ-expanded proteins (LaFevre-Bernt and Ellerby, 2003). PolyQ-expanded HTT is cleaved by caspase 2, 3, 6, and 7 as well as calpain 1 and 2, which generate toxic amino-terminal fragments containing the expanded polyQ tract (Wellington et al., 1998, 2000; Gafni and Ellerby, 2002; Gafni et al., 2004; Hermel et al., 2004). Mice expressing an HTT variant that cannot be modified by caspase 6 are protected from neurodegeneration, indicating that caspase 6-mediated proteolytic cleavage of polyQ-expanded HTT contributes to disease pathogenesis (Graham et al., 2006). Proteolytic cleavage is modulated by phosphorylation. PolyQ-expanded HTT phosphorylation at S434 by CDK5 and S536 reduces cleavage by caspases and calpains and attenuates toxicity (Luo et al., 2005; Schilling et al., 2006). Proteolytic cleavage of polyQ-expanded atrophin-1 at aspartate 109 by caspases results in aggregate formation and toxicity (Ellerby et al., 1999a; Poukka et al., 2000). Proteolytic cleavage of ataxin-3 and ataxin-7 by caspases results in protein aggregation and neurotoxicity, and mutation of the caspase cleavage sites attenuates aggregation and neurodegeneration (Garden et al., 2002; Berke et al., 2004; Young et al., 2007).

### TDP-43 proteinopathies

TDP-43 is cleaved by cellular proteases to generate carboxyl terminal fragments that are prone to aggregate and cause toxicity (Neumann et al., 2006; Zhang et al., 2009). Knocking down progranulin, a protein mutated in FTLD-U, causes caspase-dependent cleavage of TDP-43 (Zhang et al., 2007), although others have shown that proteolytic processing of TDP-43 occurs independently of progranulin (Dormann et al., 2009). Proteolytic cleavage of TDP-43 is enhanced in mice expressing mutant SOD1, suggesting its role in SOD1-linked ALS (Cai et al., 2015).

## Concluding remarks and therapeutic perspectives

The literature revised here shows that PTMs modulate several aspects of motor neuron and polyQ disease pathogenesis. PTMs affect the turnover, the degradation, and the aggregation of proteins linked to neurodegenerative diseases. Since these disorders are often caused by proteins that acquire toxic gain of function properties, PTMs that affect disease protein homeostasis are expected to modify disease outcome as well. For instance, PTMs can suppress toxicity when reducing protein stabilization and inducing protein clearance, which is often associated with reduced aggregation and inclusion formation. On the other hand, PTMs can enhance toxicity when promoting protein stabilization and accumulation inside as well as outside the cells. A key aspect in the impact of PTMs on disease pathogenesis is the effect on protein homeostasis. Indeed, when a disease protein is directly modified at the post-translational level, modulation of the activity of the enzymes responsible for such PTMs is expected to have a specific effect on disease outcome. Shedding light onto the functional role of PTMs on protein homeostasis is therefore relevant to a better understanding of the mechanisms underlying disease pathogenesis and for translating this information to therapy development. PTMs are tightly regulated, and this control results from the balance between the enzymes catalyzing a specific reaction and those catalyzing the reverse reaction. Alterations of this balance can occur in pathological conditions and can contribute to disease pathogenesis. For instance, altered Akt signaling is likely to contribute to HD and SBMA pathogenesis (Humbert et al., 2002; Rocchi et al., 2016). TDP-43 is hyperphosphorylated, ubiquitinated and subjected to proteolytic cleavage in disease conditions (Neumann et al., 2006). Protein acetylation is altered in polyQ diseases due to sequestration of proteins with HAT activity, such as CBP, into polyQ-positive inclusions (McCampbell et al., 2000; Nucifora et al., 2001; Taylor et al., 2003). Caspase activity is increased in polyQ diseases, resulting in apoptosis and aberrant proteolytic cleavage of the disease proteins (Ona et al., 1999; Sanchez et al., 1999). Another important aspect is the interplay between different PTMs. Phosphorylation of the disease proteins at specific sites affects several other PTMs, such as arginine methylation, ubiquitination, and proteolytic cleavage. Therefore, alterations in one PTM may affect other PTMs with major consequences on protein function, stability, degradation, and toxicity.

PTMs are modulated by intrinsic and extrinsic cellular signaling pathways. Activation of growth factor, neurotrophin, and their receptors can trigger signaling cascades that culminate in the activation/repression of those enzymes responsible for direct PTMs of the disease proteins. Modulation of these signaling pathways offers therapeutic opportunities. A strategy to fight the neurodegenerative process by targeting the disease proteins at the post-translational level consists in the identification of signaling pathways that induce a PTM suppressing toxicity, or alternatively in silencing signaling pathways that induce a PTM enhancing toxicity. This approach has been undertaken successfully in animal models of motor neuron and polyQ diseases, and may soon be brought to the clinic for therapy development. For instance, phosphorylation is a valuable therapeutic target. Indeed, the activity of cellular kinases and phosphatases can be pharmacologically modulated by drugs that activate and inhibit these enzymes. The observation that specific phosphorylation events diminish the toxicity of the disease proteins causing neuronal dysfunction has provided the rationale for testing the efficacy of compounds that modulate these phosphorylation events and for the development of new drugs with proven efficacy in cell and animal models of disease. Activation of Akt by the insulin-like growth factor 1 (IGF-1) signaling protects HD and SBMA cells and mice from neurodegeneration (Humbert et al., 2002; Palazzolo et al., 2009; Rinaldi et al., 2012). Therefore, IGF-1 represents a good candidate for therapy development for HD and SBMA and possibly for other neurodegenerative diseases caused by proteins that are substrate of Akt. Another strategy targeting AR by means of phosphorylation consists in the use of neuropeptides, such as pituitary adenylate cyclase-activating polypeptide (PACAP). PACAP activates the adenylyl cyclase (AC)/PKA pathway, which in turn inhibits CDK2 and reduces phosphorylation of polyQ-AR at S96, leading to protein degradation through the UPS (Polanco et al., 2016). Acetylation is a key PTM that affects polyQ and motor neuron disease pathogenesis, and as such several approaches have been developed to target HAT and HDAC activity with the aim to developing effective therapy for these disorders (reviewed by Mai et al., 2009). The HDAC inhibitors sodium butyrate, suberoylanilide hydroxamic acid (vorinostat), and phenylbutyrate ameliorated the phenotype of animal models of HD, DRPLA, and SBMA (Ferrante et al., 2003; Hockly et al., 2003; Minamiyama et al., 2004; Gardian et al., 2005; Ying et al., 2006). Similar to phosphorylation and acetylation, the observation that an increasing number of proteins linked to motor neuron and polyQ diseases are SUMOylated suggests that SUMOylation can have a disease-specific effect with therapeutic relevance. Although typically a small fraction of a protein is SUMOylated in the cells, the remarkable effects of SUMOylation of the disease proteins on toxicity suggest that this PTM is important in disease pathogenesis and may represent a good target for therapy development. Proteolytic cleavage and caspase activity are important therapeutic target. Treatment of HD mice with the tetracycline derivative caspase inhibitor, minocycline, ameliorated their phenotype (Chen et al., 2000). Also, targeting caspase activity may produce beneficial effects due to reduced proteolytic cleavage of the disease proteins and inhibition of apoptosis. Several therapeutic strategies have been pursued to target the disease proteins at the post-translational level. Evidence of beneficial effects of these experimental approaches has been obtained in animal models of disease, and efficacy in clinical trials is currently being tested.

## Author contributions

FS revised literature, wrote the paper, prepared figures and tables. MP revised literature, wrote the paper, revised figures and tables.

## Funding

This work was supported by the Muscular Dystrophy Association (479363), Telethon-Italy and Provincia Autonoma di Trento-Italy (TCP12013), Bando Progetti Strategici di Ateneo-University of Trento, Italian Ministry of Health (RF-2011-02350097), and Association Française contre les Myopathies (18722).

### Conflict of interest statement

The authors declare that the research was conducted in the absence of any commercial or financial relationships that could be construed as a potential conflict of interest.
